# Multi-Scale Stochastic Simulation of Diffusion-Coupled Agents and Its Application to Cell Culture Simulation

**DOI:** 10.1371/journal.pone.0029298

**Published:** 2011-12-21

**Authors:** Yishai Shimoni, German Nudelman, Fernand Hayot, Stuart C. Sealfon

**Affiliations:** 1 Department of Neurology and Center for Translational Systems Biology, Mount Sinai School of Medicine, New York, New York, United States of America; 2 Center for Computational Biology and Bioinformatics (C2B2), Columbia University, New York, New York, United States of America; University of Milano-Bicocca, Italy

## Abstract

Many biological systems consist of multiple cells that interact by secretion and binding of diffusing molecules, thus coordinating responses across cells. Techniques for simulating systems coupling extracellular and intracellular processes are very limited. Here we present an efficient method to stochastically simulate diffusion processes, which at the same time allows synchronization between internal and external cellular conditions through a modification of Gillespie's chemical reaction algorithm. Individual cells are simulated as independent agents, and each cell accurately reacts to changes in its local environment affected by diffusing molecules. Such a simulation provides time-scale separation between the intra-cellular and extra-cellular processes. We use our methodology to study how human monocyte-derived dendritic cells alert neighboring cells about viral infection using diffusing interferon molecules. A subpopulation of the infected cells reacts early to the infection and secretes interferon into the extra-cellular medium, which helps activate other cells. Findings predicted by our simulation and confirmed by experimental results suggest that the early activation is largely independent of the fraction of infected cells and is thus both sensitive and robust. The concordance with the experimental results supports the value of our method for overcoming the challenges of accurately simulating multiscale biological signaling systems.

## Introduction

Gene expression and signaling events in single cells are stochastic processes. Population measurements and simulations that reflect average cellular responses obscure many aspects of cellular dynamics [1]–[7]. When stochastic processes are considered, analyses of single cell systems are normally performed assuming that the cells do not interact, and simulations are done using either Gillespie's algorithm [8], or Langevin equations [9]. Gillespie simulations follow the number of molecules present in a cell for several molecular species, and quickly become inefficient as the number of molecules becomes large. Langevin equations can be solved efficiently numerically, but can only be used accurately under a set of restrictive assumptions. Several modifications to the classical Gillespie algorithm have been proposed, some of which lead to more efficient computation [10]–[13]; others offer parallelization of the algorithm [14], [15], and others separation of time scales through the use of hybrid deterministic-stochastic approaches [16], [17], or by allowing to estimate the effect of processes that may occur multiple times during a time-step (also known as tau-leaping) [18].

All the techniques mentioned above assume that the cell's state can only change due to processes explicitly defined within the simulation framework. Therefore, when multiple interacting cells are simulated, all cells must be incorporated into a single large simulation, in which identical processes in different cells are counted as different processes. When cells are close to each other, or if the interaction between them is direct in some other way, an efficient compartmental model can be used by including the volume immediately surrounding each cell in the simulation [19]. Such simulations, however, must account for each and every molecule that passes from one compartment to the next, and become unfeasible for large concentrations of signaling molecules.

The computational problem is compounded when the signaling molecules diffuse in the medium and are not directly transferred from cell to cell, since the volume between the cells must be divided into more and more compartments. One way to deal with this problem is to use agent-based models (ABMs), where many individual cells are simulated using the same set of rules (where each agent is a simulation of a single cell). The simulation allows each cell to interact with its local environment. In this case the diffusion process can be simulated separately from intracellular dynamics, allowing separation of time-scales between the internal and external processes.

Such an ABM model was recently developed, in which independent stochastic agents were simulated in conjunction with diffusion processes [20]. However, this work used a deterministic solution to the diffusion equation for cell to cell signaling. Thus, the simulation and resulting analysis ignored the stochastic nature of the interaction between cells. At high concentrations stochasticity may not cause significant variations, but when small concentrations of molecules are involved, an explicitly stochastic approach must be used. Such a simulation, however, that follows the movement of every single molecule is computationally impractical.

Multiple techniques were recently developed to efficiently simulate stochastic reaction-diffusion prcoesses [21]–[23]. These techniques utilize the statistical properties of independent diffusion processes to allow multiple events of diffusion to occur in each time step (tau-leaping). Notably, one of the methods [24] employs a similar solution to the one we propose here, and may also be applicable to the system we analyze. The solution presented in [24], however, is more complicated conceptually, and more elaborate to implement.

Here we present a method than can be used to stochastically simulate a population of cells that interact by exchanging mediators in a diffusive manner. We first introduce an algorithm that is used to efficiently and stochastically simulate diffusion, and is based on the Monte-Carlo approach. We show that the algorithm leads to random walk behavior for low concentrations, and to dynamics identical to those obtained from the diffusion equation for high concentrations. A second algorithm is introduced, which is a modification of the Gillespie algorithm, and is used to stochastically simulate the internal dynamics of individual cells in a way that ensures a behavior suitable for a Markovian process while allowing changes in external conditions. Diffusing molecules that bind to cell surface receptors activate signaling pathways inside the corresponding cells. The challenge is to match the dynamical intracellular updating as given by Gillespie's algorithm (which is of the order of several minutes), to the time scale associated with diffusion and binding in the extra-cellular medium (which is of the order of several seconds). The modification introduced in the algorithm allows synchronization between the diffusion simulation and the simulation of individual cells at preset time intervals, and is a natural consequence of the Markovian assumption. For convenience we refer to this algorithm as the synchronized Gillespie algorithm. Applying the stochastic diffusion algorithm and the synchronized Gillespie algorithm jointly in an ABM provides an accurate stochastic simulation method for a culture of cells which interact through the diffusion of molecules. As required, this method allows a separation of the time scales between the stochastic diffusion simulation and the synchronized Gillespie algorithm, since the time scales in each one is independent from the other, thus creating a multi-scale simulation.

**Figure 1:**
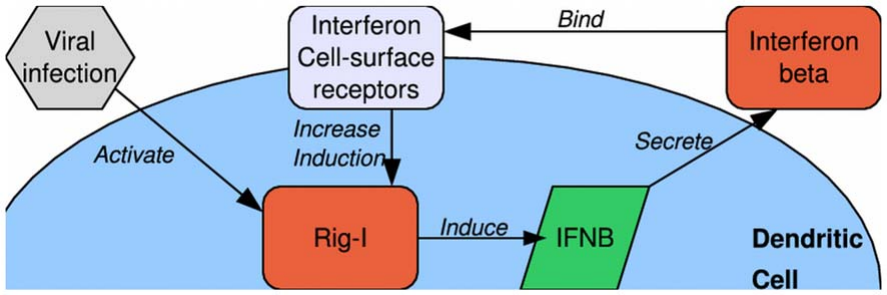
Schematics explaining the positive feedback loop between Rig-I and interferon beta in infected cells. Viral infection is detected by basal levels of Rig-I proteins, which become activated and induce transcription and subsequent secretion of interferon beta. Interferon beta molecules that bind to cell-surface receptors enhance the transcription of Rig-I proteins, thus either completing or activating the positive feedback loop.

Specifically, the proposed method allowed us to simulate a culture of monocyte-derived human dendritic cells (DCs), which are the primary response cells mediating the progression from innate to adaptive immunity [25], [26]. When a virus such as the Influenza A virus or Newcastle Disease virus infects a DC, the DC is able to detect the infection using the Rig-I protein [27]. This causes the cell to secrete various chemokines and cytokines, of which the most important is interferon. When interferon binds to cell-surface receptors on DCs, it activates the transcription of a large number of genes, including the gene that encodes Rig-I, thereby completing a positive feedback loop that enhances the DCs response to viral infection [28]. A schematics of the mechanism is shown in Fig. 1. Using our simulation method we have recently shown that only a small subset of the cells recognize the viral infection at early times (early responders), and that these cells signal the other DCs and alert them by secreting amounts of interferon that are undetectable experimentally [29]. This small subset is hard to distinguish from experimental noise, and thus simulations become the appropriate method of investigation of this system. Since only a small subset of a population of identical cells cause the response, accurate simulation requires stochastic simulation of individual cells. Additionally, in order to capture the effects of local interferon concentrations at early times, diffusion processes must also be handled stochastically, with allowance made for the high concentrations measured experimentally at longer times following infection. Thus to accurately simulate this system a simulation procedure such as the one proposed is required.

Although DCs are known to adjust the magnitude of response to viral load, the relation of the sensitivity and the control mechanisms to multiplicity of infection (MOI), which is the ratio between the number of viruses and the number of cells in the culture, remains unclear. Here we use our algorithm to investigate how the spreading of cellular response to viral infection depends on MOI. We show that all the infected cells can be activated with similar timing with any MOI of 0.5 or higher, leading to similar interferon production levels. This allows a highly sensitive response to viral infection, which is robust, controlled, and reliable. The results presented here exemplify the value of algorithms for simulations of large systems in which stochastic agents interact via a diffusive process.

## Methods

### Stochastic Spatially Explicit Diffusion Algorithm

Stochastic simulation of diffusion processes are challenging since they must account for the positions of large numbers of molecules, requiring both large amounts of memory, and strong computational power. One solution to this problem is to consider the local concentration of molecules instead of the location of individual molecules, and follow their dynamics using the diffusion equation. Here, the simulation follows the local concentration by dividing the space into voxels (or grid squares in 2D), and following the number of diffusing molecules 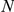 per grid square. Using an optimized Monte Carlo algorithm, this allows the underlying random walk behavior of individual molecules while allowing efficient simulation with memory costs that do not depend on the number of molecules in the simulation.

For simplicity we introduce the algorithm in 2D, and later discuss its extension to 3D. We limit the discussion to the experimental scenario described in section I. It should be noted, however, that the simulation can be performed for any set of stochastic entities that interact using secretion and binding of diffusing molecules.

Consider a system in which the agents in the simulation are cells in a medium which we represent as a square lattice. The cells are randomly distributed in the lattice, and periodic boundary conditions are used. We choose the lattice square size to be the size of a single DC, with characteristic length 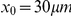. Thus, each grid square can either contain a single cell or no cell, and conversely, each cell occupies a single lattice square and does not move throughout the simulation. Additionally, each grid square contains a number of interferon molecules, that can diffuse to neighboring lattice squares with a diffusion coefficient 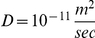 that was determined experimentally [30]. The diffusion algorithm itself is iterative, with each iteration consisting of two steps for each lattice square:

- For each molecule, decide randomly with a probability 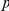 whether within the next time-step it diffuses to a neighboring lattice square.
- For each diffusing molecule, choose with equal probability the direction in which it diffuses, and move it to the appropriate neighboring square.

As presented above, the algorithm is not efficient since a random number has to be generated for each molecule, which is essentially equivalent to following the movement of every single molecule. To avoid this computationally taxing procedure we reformulate the steps for each grid square as follows:

- Choose the number of diffusing molecules according to a binomial distribution 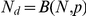.
- Out of the diffusing molecules choose the number of molecules that diffuse north and south according to a binomial distribution 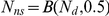. The rest of the diffusing molecules will diffuse east and west, giving 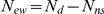.
- Out of 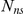 choose the number of molecules that diffuse north according to a binomial distribution 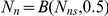, thus the number of molecules diffusing south is 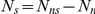.
- Out of 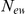 choose the number of molecules that diffuse east according to a binomial distribution 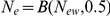, and so the number of molecules diffusing west is 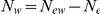.

This strategy eliminates the need to follow each molecule by choosing a random number for it, and sets the number of random numbers at a constant 4 per lattice square. However, choosing a number from the binomial distribution 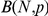 requires computation time that still depends linearly on the number of molecules 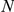. A simple way to make this process much more efficient is to build a lookup table for the cumulative binomial distribution 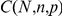. This lookup table holds 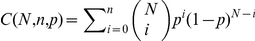 for every 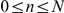 and for every 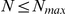, where 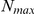 is the maximal number of molecules found in any lattice square. Thus, a random number can be chosen from a uniform distribution between 0 and 1, and the appropriate location in the sorted lookup table can be efficiently found using a binary search. Using such a lookup table, the execution time of the algorithm scales like 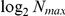, and scales linearly with the area of the simulated system. More accurately, the run-time of each step depends on the sum 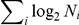, where 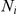 is the local concentration in grid square 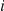. It is reasonable to choose 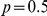, so that a single lookup table can be used both for choosing the number of diffusing molecules and for choosing the number of molecules diffusing in each direction.

It should be noted that relatively efficient approximation methods are available to compute the cumulative probability distribution function for the binomial distribution for any choice of 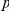 [31]. The approximation presented in Ref. [31], however, scales like 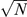 and is therefore less efficient than using a lookup table. For large numbers the binomial distribution 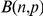 can be approximated by a normal distribution with an average 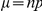 and variance 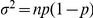, which can be calculated efficiently using many existing libraries.

The algorithm to simulate diffusion in 3D can be easily generalized from the 2D procedure presented above. Here, the number of diffusing molecules in each lattice cube will be chosen with some probability 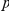, similar to the 2D case. After choosing the number of diffusing molecules one of six directions has to be chosen. Five random numbers must be chosen for this (e.g. two to choose between axes X, Y, and Z, and one to choose the sign in each axis). A straightforward implementation of this algorithm requires two lookup tables, one for the binomial distribution 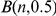 and the other for 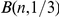. This slightly increases the memory requirements of the algorithm, but retains the property that the computation time scales like 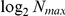, and linearly with the volume of the simulated system. Alternatively, one can use the lookup table with 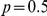 to choose between axis X, Y, Z, and R, (where R stands for ‘repeat’), and repeat the process for R iteratively until the number of molecules in direction R is zero. This is a potentially infinite procedure, but on average would require 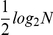 iterations, and so the total average algorithm run-times will scale like 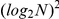, while only a single lookup table is required.

### Selecting Appropriate Time Step and Lattice Square Size

When simulating diffusion processes, the diffusion coefficient 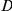 determines a connection between the time interval 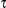 and the grid size. Normally, one of the parameters (i.e. grid size or time interval) is determined using some external considerations. In our example the lattice grid size was chosen according to the average cell length 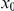. To determine the other parameter, the dimensionality of the diffusion coefficient is used to obtain 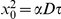, where 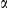 is an unknown multiplicative constant that depends on 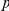. For the simulation to be accurate it is necessary to obtain the correct value for 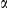.

**Figure 2:**
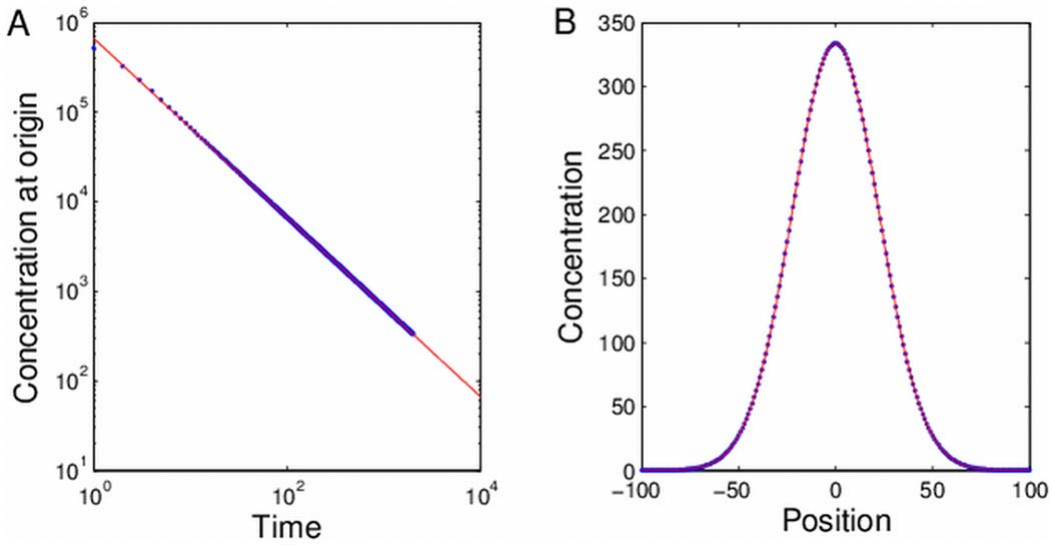
Fitting simulation results to analytical solution. Results of a deterministic diffusion simulation in which at each time step half the molecules at each grid square are equally distributed to neighboring squares. 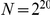 molecules were placed in the middle of the grid at time 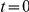. A. The concentration in the middle of the grid as a function of simulation steps (dots) and a fit to the inverse of time (solid line). B. The concentration in a horizontal section through the middle of the grid after 2000 simulation steps (dots), and a fit to a Gaussian function (solid line).

This was done by fitting the diffusion simulation algorithm described above to an analytical solution of the diffusion equation, as shown in Fig. 2. Consider the analytically solvable case where at time 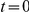 all the molecules are located at the origin (using the Kronecker delta function). We initialized the simulation with all the molecules (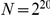) concentrated at the middle of a 201 by 201 lattice, and ran the simulation for 1000 time steps. Due to the high number of molecules stochastic effects are negligible, and therefore at each time step we moved exactly one half of the molecules in each grid square, and distributed them equally in all four directions. For the simulation to be considered accurate, the concentration profile of the simulation must follow the solution of the diffusion equation for this condition, which is given by
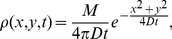
(1)where 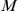 is the total amount of molecules in the system.

Fig. 2A shows the concentration at the origin as a function of simulation time in a log-log plot (dots), and a fitted 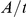 function (solid line), where 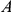 is the fitted parameter. Except for several steps at the beginning of the simulation, the fitted functions shows a remarkable fit (r-square 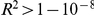). Fig. 2B shows the concentration of a section through the origin at the end of the simulation (dots), and a fitted Gaussian (solid line), and shows an exceptional fit (r-square 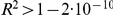).

The fit presented in Fig. 2A not only shows that the simulation produces the correct dynamics in the functional sense, but also suggests a means to find the appropriate time-length 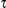 to be used in the simulation (given the size of the lattice square 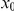 and 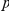). Comparing the fitted function 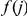 to the required solution we get
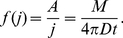
(2)where 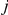 is the number of simulation steps. Since 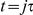, we immediately get that 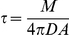. 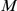 and 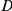 are known and 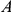 is given by the fit, and so 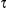 can be extracted.

The fit presented above provides an accurate relation between simulation time and real time using the specific values for 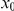 and 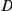 presented in our system. This can be generalized by remembering that the simulation is independent of 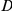 and 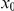 and only depends of 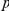, so we can derive the multiplicative constant 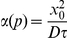. By substituting the values for 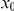, 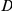, and 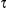 we get 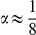, or 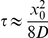. We note that this result is true only for a 2D simulation with 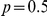. However, the correct coefficient can be easily found in a similar fashion for any value of 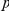, as well as for a 3D simulation. Specifically, for 3D and 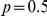 we get 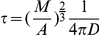, and by performing a simulation and fitting the resulting function we get 
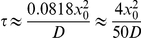
.

### Determining the Fraction of Diffusing Molecules

In some cases both the time step 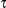 and the lattice size 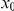 are determined from other considerations. Under such circumstances it is necessary to determine 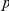, the average percentage of molecules that diffuse out of a lattice square within a single time step. The procedure presented in the previous section does not provide a solution for this case, since Eq. 1 provides a relation between 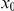 and 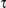, but not 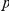. An approximate relation between all three variables can be derived by assuming that at time 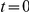 all the molecules are concentrated in the middle of a grid square, and integrating over the solution to the diffusion equation across the lattice square at time 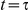. This gives the proportion of the mass that remains in a grid square after time 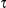, which is 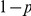. Mathematically, this means solving the equation
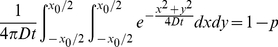
(3)for 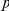. Using the symmetry of the problem and substituting the integration variable the equation can be re-written in the form
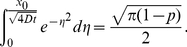
(4)This integral is known as the error function (erf) and cannot be solved analytically, but can easily be solved numerically to find 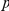 (and is available as a built-in function in most mathematical libraries).

We note that there are two approximations involved in this analysis. The first is that in the simulation we assume that the molecules are equally distributed (well-mixed) within each grid square, while the mathematical analysis assumes that they are all concentrated at the middle of the square at the start of every time step. The second approximation is that the simulation only allows molecules to diffuse to a neighboring cell at each time step, while the mathematical analysis allows a small percentage to diffuse farther away at that time. In agreement with this analysis, substituting the values found above for 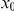 and for 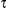 in Eq. 4, we find a value for 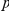 that is close to 0.5 (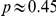). In order to find the accurate value of 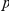 simulations are needed in which the value of 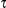 (or 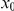) is fixed. As explained in the previous subsections, the values of 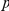 and 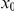 determine the time step 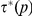 that must be used in the simulation (or 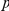 and 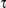 determine the size of the lattice square 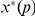). Using half-interval search a value of 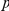 can be efficiently found up to arbitrary precision so that 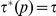 (or 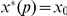).

### The Synchronized Gillespie Algorithm

The Gillespie algorithm is a simple algorithm that allows accurate simulation of a Markovian process [8]. The algorithm follows the number of molecules for 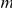 molecular species involved in 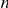 processes. The rate at which process 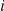 (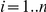) occurs is given by 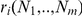, where 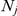 (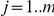) is the number of molecules of type 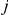. The vector 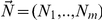 represents the state of the system, and the vector 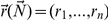 is the vector holding the transition rates. The fact that the rates of transition between states depend solely on the state itself and not on the simulation history makes it a Markovian process. The algorithm is an iterative one, where at each simulation step the following procedure is performed:

- Choose the time 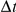 until the occurrence of the next process from an exponential distribution with an average 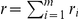, and advance time by 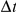.
- Choose randomly which process occurs, where each process has a probability proportional to its rate.

The Gillespie simulation results in a different trajectory in phase space at each execution. Still, the statistics of these trajectories are identical to the solution of the master equations for the same system, which are equations describing the dynamics of the probability to find the system in any given state at any given time.

The Gillespie algorithm, however, assumes that the state of the system 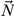 and the transition rates 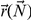 can only be changed by the algorithm. Namely, it assumes that every process that can change 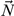 or 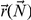 is described by the Gillespie algorithm itself, and not by some other algorithm running in parallel to it. Although it is theoretically possible to incorporate every process within the same Gillespie simulation, such a simulation would become increasingly more complex and inefficient. When integrating a Gillespie simulation into an ABM that allows interactions between agents, the state of the system will invariably be changed by other agents or the environment.

For example, in our case diffusion (which is part of the ABM) changes the number of bound interferon receptors, which affects the virus detection capabilities of the cell through the rate of Rig-I production. In the context of the Gillespie simulation of each cell, the diffusion simulation changes 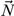 externally at constant intervals, thus breaking the basic assumption of the Markovian process. To account for this, the synchronized Gillespie simulation stops if it tries to update 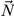 or 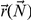 after the time at which 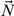 is supposed to be changed externally (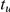), and advances its internal time to 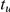. It then allows the external update of 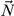 and the resulting rates, and continues from that point on using the newly updated rates. This translates to a modification in the Gillespie updating procedure at each simulation step:

- Choose the time until the occurrence 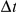 of the next process from an exponential distribution with an average 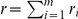.
- If the expected time for the next step is larger than the next external state update time 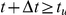 then advance the time to 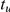 and allow the external state update. Otherwise advance time by 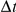, and choose which process occurs, where each process has a probability proportional to its rate.

This modification is an immediate consequence of the Markovian assumption that is at the heart of the algorithm. To show this, we briefly explain the derivation of the exponential distribution for the time-step in the original Gillespie simulation, and then proceed to explain the validity of the modification. We start by assuming that the rate 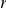 of a process depends only on the state of the system. As a result, until any process occurs the probability of that process occurring within the next short time interval 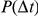 is constant. Therefore, by dividing a long time interval 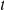 into 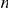 short intervals the probability of nothing happening within 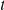 can be written as
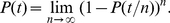
(5)Since 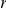 is the derivative of 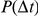 according to time, for short enough time intervals 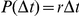, and so Eq. 5 can be rewritten as
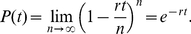
(6)


Similarly, consider the case where at a certain time 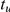 the state of the system changes so that the rate of the process changes to 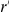. For 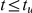 the analysis remains as presented above, but for 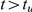 the probability 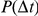 changes, and so we get
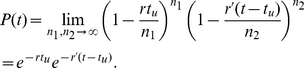
(7)Therefore, an initial time 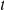 at which the process may occur should be drawn from an exponential distribution with an average of 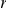, as before. If the time 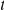 is smaller than 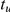 then this time should be used. However, if 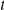 is larger than 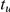 (which happens with probability 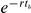) then a new time 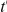 should be chosen, independendt of 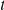, from an exponential distribution with an average 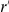, and the time at which the process occurs is then 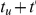. This procedure is reflected precisely in the proposed modification to the Gillespie algorithm. We note that this method can be considered a simplified special case of a method presented in [18].

**Figure 3:**
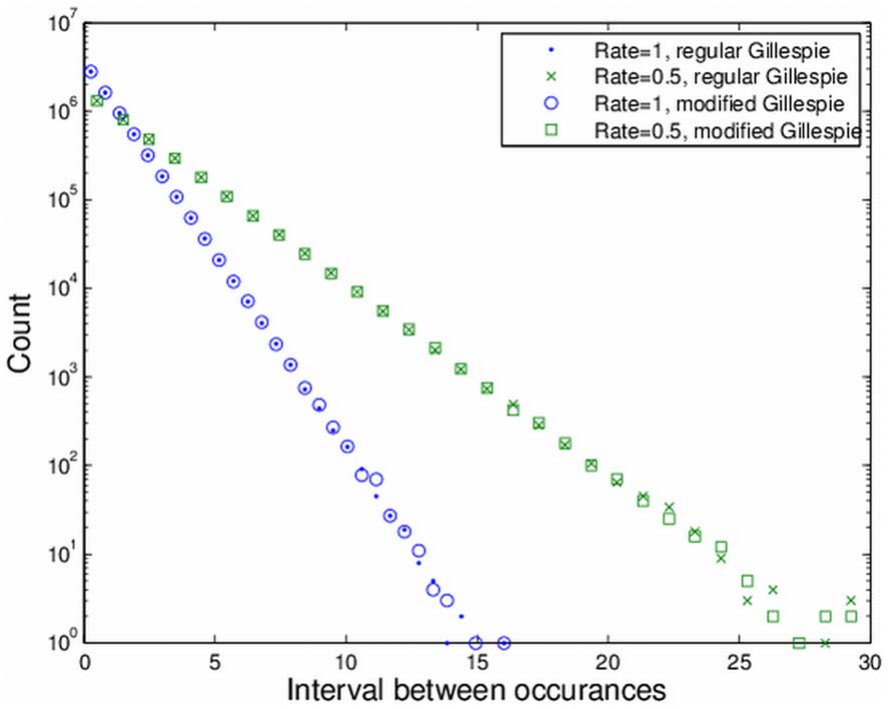
Consistent behavior of the synchronized Gillespie algorithm. Histograms of the intervals between consecutive occurrences of processes in two stochastic simulations. Both simulation were performed with two processes whose rates do not change during the simulation. The first simulation used the original Gillespie algorithm (dots and X's), and the second one used the modified Gillespie algorithm (open circles and open squares) without changing the state of the system during synchronization. Both processes display the same exponential distribution with the correct mean.

To ensure that this procedure does not introduce artifacts we performed a synchronized Gillespie simulation where at each synchronization step the algorithm stops but no change is made to the state of the simulated system. The times between successive occurrences of the process in such a simulation should display a distribution that is identical to the original Gillespie simulation. Fig. 3 shows such histograms derived from two simulations involving two processes with rates 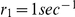 and 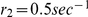. The first simulation used the original Gillespie algorithm (dots and X's), and the second one used the synchronized Gillespie algorithm (open circles and open squares). The rates were not changed throughout the simulations, which were performed to a total of 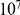 process occurrences. Both simulations result in identical exponential distribution with the correct mean time between occurrences, showing that the procedure is accurate.

### Simulation parameters

Simulation parameters were the same as those previously reported [29]. Below we give a brief description. The modified Gillespie simulation for internal cell dynamics follows the time dependence of interferon transcript (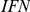), Rig-I transcripts (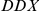) and Rig-I proteins (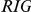), which are involved in six reactions: 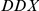 transcription, 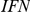 transcription, 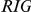 translation, 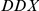 degradation, 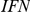 degradation, and 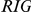 degradation. The rate constants for maximal 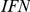 and 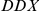 transcription are given by 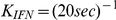 and 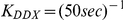, respectively. The translation rate constant for 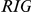 is given by 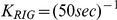. The degradation rate constants for 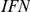, 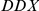, and 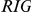 are given by 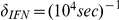, 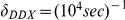, and 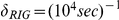, respectively. The transcription rate of Rig-I transcripts depends on the number of bound interferon receptors, denoted by 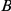, in a Michaelis-Menten form using a Hill coefficient of 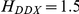 and a half-induction level given by 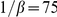 (meaning that when 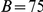 Rig-I reaches half it maximal induction). According to experiments, Rig-I is constitutively expressed in the cells. The ratio between the maximal induction of Rig-I and the constitutive induction is given by 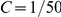. Interferon induction depends on Rig-I concentration in a Michaelis-Menten form with a Hill coefficient 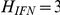, and a half-induction concentration given by 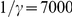. All the degradation processes follow an exponential decay.

For the diffusion simulation we use a two-dimensional 40 by 40 square lattice with a square-side length of 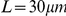 (which is the average diameter of a cell ,as observed experimentally), and holding 200 randomly distributed cells. The diffusion coefficient was determined experimentally to be 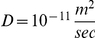 [30]. The diffusion time step was determined (as explained above) to be 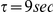. At each grid square the simulation follows the number of free interferon molecules 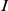, and in squares containing cells also the number of bound receptors 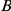 and free receptors 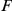. The initial number of free receptors is 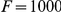 per cell. At each time step interferon molecules in lattice squares containing cells may bind to free surface receptors, while bound receptors may unbind. The binding rate constant is given by 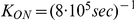, and the unbinding rate constant is 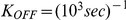. We assume that the number of new interferon molecules that are translated at each time step is the number of 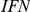 transcript multiplied by 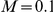. We further assume that interferon secretion is rapid and that all the newly synthesized molecules are secreted at each time step.

## Results

**Figure 4:**
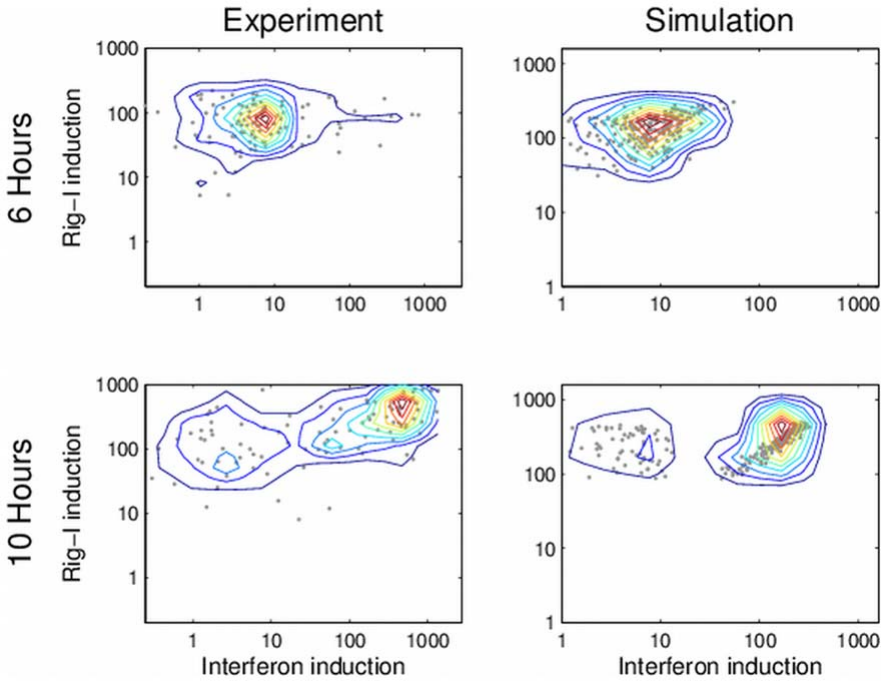
Comparison between experimental results and simulation results of the transcription induction of Rig-I and of interferon. The panels show the amount in individual cells (dots), and an interpolated contour plot of the 2D histogram (solid lines). The two left panels show the experimental results and the two right panels show simulation results. The two top panels and two bottom panels show results obtained at 6 hours and 10 hours, respectively. Significant similarity can be seen between the experimental results and the simulation results. A small population of early responders can be seen in the simulation results at 6 hours, corroborating the early responders hypothesis.

We used the proposed ABM simulation to investigate the response to viral infection of monocyte-derived human dendritic cells (DCs), which are the primary response cells that detect infection and trigger the initial innate immune response [25], [26]. As depicted in Fig. 1, during the innate immune response, DCs detect viral infection using several receptors and proteins, one of which is Rig-I. Activated Rig-I molecules set in motion a signaling cascade that leads to the induction of the interferon beta gene, which upon translation into protein is secreted from the cell. Interferon molecules bind to cell-surface receptors on both infected and uninfected DCs, activating a host of interferon-induced genes, among which is the gene coding for Rig-I. These interactions constitute a positive feedback loop between interferon beta and Rig-I. We recently showed that a fraction of the DCs (less than 1%, the early responder cells) which have sufficient levels of constitutive Rig-I initiate interferon transcription. These cells secrete large amounts of interferon that create locally high concentrations and activate neighboring cells, which in turn produce elevated levels of Rig-I, and if infected subsequently produce and secrete high amount of interferon as well. [29] The ABM proposed here presents a suitable approach to follow the dynamics of the system in a reliable manner, and allows an understanding of the effects of stochasticity on inter-cellular interferon signaling. Fig. 4 shows the levels of Rig-I mRNA vs. interferon mRNA in individual cells, as obtained either from experimental results [29] (left) and from simulation (right) at 6 hrs after infection (top) and 10 hours after infection (bottom).

**Figure 5:**
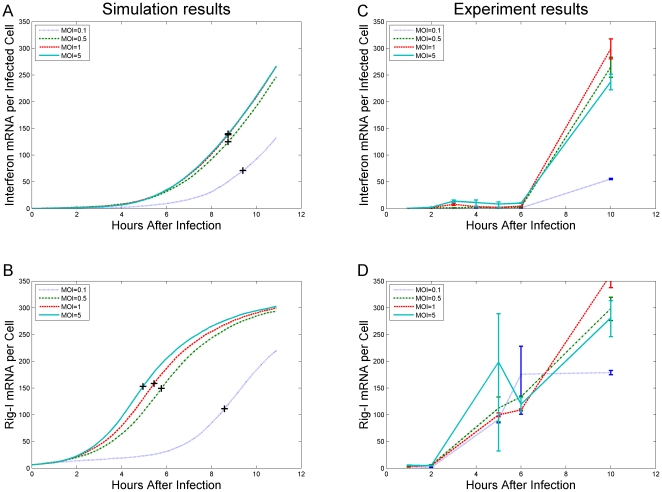
Simulated time-courses of average concentrations for several MOIs and corresponding experimental results. The lines represent the average concentrations of interferon transcripts (panels A and C), and Rig-I transcripts (panels B and D) as obtained by simulation (panels A and B) or from experiments (panels C and D). Both the simulations and the experiments were performed with MOI = 0.1 (dotted lines), MOI = 0.5 (dash-dotted lines), MOI = 1 (dashed lines), and MOI = 5 (solid lines). Specifically, panel A displays the number of interferon mRNA molecules per infected cell as a function of time as predicted by simulation, and panel B presents the number of Rig-I mRNA molecules per cell as predicted by simulation. The time and value of half of the maximal induction is marked by plus markers. Panels C and D show experimental results corresponding to the simulation predictions in panels A and B, respectively. MOI = 0.5, MOI = 1, and MOI = 5 exhibit very similar dynamics, suggesting the activation mechanism is both sensitive and controlled.

Although the early response mechanism enables a sensitive yet controlled response to viral infection, the effect of MOI on the early activation of the small sub-population of cells remains unclear. In order to explore the important aspect of robustness of the immune response with changing levels of the multiplicity of infection (MOI), we expanded the previous analysis and performed simulations with varying levels of MOI. In these simulations we examined the efficiency of the immune response activation by the early responder cells. Fig. 5A and Fig. 5B show the levels of interferon and Rig-I mRNA (respectively) as predicted by simulations with MOI = 0.1, 0.5, 1, and 5, which can be compared to the experimental results shown in Fig. 5C and Fig. 5D (the experimental results redrawn from reference [29]). As expected, in a simulation with MOI = 0.1 (dotted lines) the number of early responders that are activated in the simulation is insufficient to efficiently activate the whole population of cells. Most of the cells in the simulation are self activated, and as a result there is hardly any difference between the time of half-maximal induction of interferon and that of the Rig-I gene, (marked by +'s in Fig. 5A and Fig. 5B). On the other hand, it is notable that the lines for MOI = 0.5, 1, and 5 (dash-dotted, dashed, and solid lines, respectively) are very similar. This result suggests that the coordinated response of the cells when activated by early responder cells is highly robust, in the sense that it is both sensitive to low levels of infection, and produces a similarly controlled response even at high levels of infection. The results of these simulation are consistent with the experimental results presented in Fig. 5C and Fig. 5D, where it is shown that increasing the MOI does not result in an increased induction of Rig-I. High noise levels in the experimental results make it difficult to estimate the goodness of the fit at early times (especially for MOI = 0.1, at 5 and 6 hours), but the final result (at 10 hours) is consistent with the simulation predictions, and suggests that the ratio of early responder cells is similar to the one used in the simulation, namely approximately one percent.

## Discussion

In this paper we introduced a methodology to combine two stochastic algorithms into an agent based model (ABM) of agents that interact via a diffusive process. The first algorithm allows accurate and efficient stochastic simulation of diffusion processes at both low and high concentrations, and the second allows stochastic simulation of a Markovian system in which the conditions are changed due to external circumstances (i.e. outside the scope of the simulation).

The stochastic diffusion algorithm was introduced and was shown to reproduce the required random walk behavior at low concentrations, and to fit the exact solution to the diffusion equation at high concentrations. The algorithm was introduced in 2D and extended to 3D. The fact that the algorithm depends on the probability 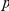 of a molecule to diffuse to a neighboring lattice point at every simulation step allows to extend the algorithm to the diffusion of multiple molecular species. To do that, each species receives its own value of 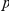. The value of 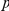 for each molecular species can be obtained in a similar way to the one presented above.

The synchronized Gillespie algorithm introduces an important modification to the original Gillespie algorithm, that allows it to accurately run in parallel to other simulations, even when both simulations affect the same molecular species. The proposed modification can be easily applied in combination with various improved versions of the Gillespie algorithm [10]–[15], allowing for even more efficient and modular simulations.

The usefulness of the combination of these algorithms was demonstrated by applying them for a culture of human DCs that alert each other to viral infection using secretion and diffusion of interferon molecules. The simulation method was previously used [29] to corroborate the hypothesis that a small sub population of the DCs are activated quickly by the infection and alert the other cells, thus expediting the overall immune response. We expanded on these results, and showed that this mechanism of activation is sensitive to small levels of infection (i.e. low MOI), but is highly controlled even for high levels of infection.

It should be noted that our method is not limited to simulation of DCs secreting interferon, or even only to cell cultures, but that it can also be applied to other fields in which agents interact using diffusion processes (e.g. biofilms). Additionally, the modification to the Gillespie algorithm enables precise stochastic simulations of systems that can be affected by external conditions (e.g. externally triggered or time dependent cell division). Finally, the combination of both algorithm allows a separation of time scales between the Gillespie simulations and the diffusion simulation. Each individual Gillespie simulation uses its own set of reaction rates, allowing for a simulation with multiple time scales.

In conclusion, the methodology presented here paves the way to more extensive analysis of stochastic processes that occur at the scale of cell culture and tissue, or that involve different time scales.
